# TRPA1 mediates the hypothermic action of acetaminophen

**DOI:** 10.1038/srep12771

**Published:** 2015-07-31

**Authors:** Clive Gentry, David A. Andersson, Stuart Bevan

**Affiliations:** 1 Wolfson Centre for Age-Related Diseases, King’s College London, London SE1 1UL, UK

## Abstract

Acetaminophen (APAP) is an effective antipyretic and one of the most commonly used analgesic drugs. Unlike antipyretic non-steroidal anti-inflammatory drugs, APAP elicits hypothermia in addition to its antipyretic effect. Here we have examined the mechanisms responsible for the hypothermic activity of APAP. Subcutaneous, but not intrathecal, administration of APAP elicited a dose dependent decrease in body temperature in wildtype mice. Hypothermia was abolished in mice pre-treated with resiniferatoxin to destroy or defunctionalize peripheral TRPV1-expressing terminals, but resistant to inhibition of cyclo-oxygenases. The hypothermic activity was independent of TRPV1 since APAP evoked hypothermia was identical in wildtype and *Trpv1*^*−/−*^ mice, and not reduced by administration of a maximally effective dose of a TRPV1 antagonist. In contrast, a TRPA1 antagonist inhibited APAP induced hypothermia and APAP was without effect on body temperature in *Trpa1*^*−/−*^ mice. In a model of yeast induced pyrexia, administration of APAP evoked a marked hypothermia in wildtype and *Trpv1*^*−/−*^ mice, but only restored normal body temperature in *Trpa1*^*−/−*^ and *Trpa1*^*−/−*^*/Trpv1*^*−/−*^ mice. We conclude that TRPA1 mediates APAP evoked hypothermia.

Acetaminophen (APAP) is a widely used analgesic and antipyretic drug, but its mechanism(s) of action has remained elusive. Although APAP may inhibit cyclo-oxygenase (Cox-1 and Cox-2) enzymes at high concentrations, this is not thought to be the primary mode of action. The lack of a strong anti-inflammatory activity is consistent with a mechanism that does not involve cyclo-oxygenase inhibition. Furthermore, APAP in combination with a COX inhibitor, such as ibuprofen, provides improved analgesia^1^ and antipyretic activity^2^ compared to either compound alone, suggesting that they act through different mechanisms.

Recent studies have led to proposals that the analgesic effects of APAP are due to actions of metabolites of the parent drug on sensory neuron TRP channels. The electrophilic APAP metabolite N-acetyl-*p*-benzoquinoneimine (NAPQI) is a potent TRPA1 agonist, which is formed in the spinal cord, in addition to the liver and kidneys^3^. Both APAP itself and its metabolite NAPQI exert an analgesic effect when administered intrathecally to wild-type mice. This analgesic effect is absent in *Trpa1*^*−/−*^ mice as is the analgesic effect of systemically administered APAP^3^. APAP can also be metabolized to form an N-acylphenolamine derivative, AM404, which is a TRPV1 agonist^4^ that produces TRPV1 dependent analgesia when administered intracerebroventricularly^5^. APAP is an effective antipyretic agent, which produces a pronounced hypothermia when administered to rodents^6^,^7^ and a small hypothermic effect in humans^8^, a difference which is probably explained by the larger surface to volume ratio and higher rate of heat loss in smaller animals.

TRPV1 activity *in vivo* regulates body temperature and TRPV1 agonists such as capsaicin and resiniferatoxin have long been known to produce hypothermia in rodents^9^,^10^,^11^, whereas pharmacological inhibition of TRPV1 evokes a marked hyperthermia in mice and humans^12^. Although TRPV1 activation could therefore underlie the hypothermic effects of APAP, studies with TRPV1 deficient mice and a TRPV1 antagonist indicated that APAP induced hypothermia was independent of TRPV1^7^. In contrast, the effect of TRPA1 activity on body temperature has not been examined in detail. Since TRPA1 is co-expressed with TRPV1 by a substantial percentage of sensory neurons, stimulation of TRPA1 in these nerve fibres would be expected to exert a similar hypothermic effect to that seen with TRPV1 agonists. A TRPA1 antagonist was reported to have no hyperthermic or hypothermic effect^13^, but a direct effect of an antagonist would rely on some degree of tonic TRPA1 activity as is thought to be the case for TRPV1^12^.

In the current study we used genetically modified mice and pharmacological tools to determine the contribution of TRPA1 to the hypothermic actions of APAP. Our data demonstrate that APAP evoked hypothermia in mice is dependent on the presence of functional TRPA1 channels. The hypothermic effect of APAP is lost in *Trpa1*^*−/−*^ mice and inhibited by administration of a TRPA1 agonist.

## Results

### Involvement of TRPA1

**Figure 1:**
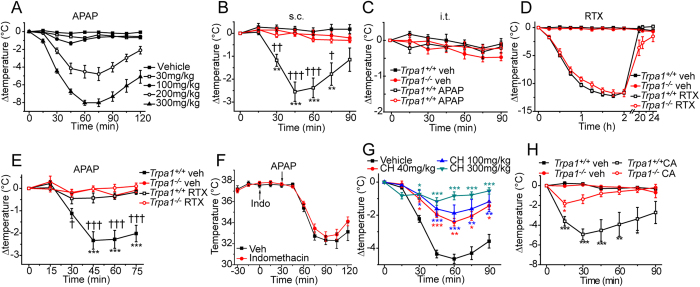
APAP elicits TRPA1-dependent hypothermia. (**A**) Subcutaneous injections of APAP evoked a dose-dependent hypothermia in C57BL/6J mice (n = 6–12). (**B**) APAP (300 mg/kg) reduced the body temperature in *Trpa1*^*+/+*^ mice but was without effect in *Trpa1*^*−/−*^ mice (n = 6–11, see panel C for key to the symbols). (**C**) Intrathecal administration of APAP (100μg) did not reduce body temperature (n = 6). (**D**) Resiniferatoxin (RTX, 6.3 μg/kg, s.c.) induced a profound hypothermia in *Trpa1*^*+/+*^ and *Trpa1*^*−/−*^ mice (n = 6). (**E**) APAP (300 mg/kg s.c.) evoked hypothermia in non-RTX treated *Trpa1*^*+/+*^ mice, but was without effect in *Trpa1*^*−/−*^ and RTX treated *Trpa1*^*+/+*^ mice (n = 6). (**F**) Indomethacin (10 mg/kg, s.c.) did not alter body temperature and did not inhibit APAP (300mg/kg) evoked hypothermia (n = 6). (**G**) Oral administration of the TRPA1 antagonist CHEM5861528 (40–300 mg/kg, 45 min before APAP) produced a dose-dependent inhibition of APAP evoked hypothermia (n = 6). (**H**) Intraperitoneal injections of the TRPA1 agonist cinnamaldehyde (CA, 125 mg/kg) induced a marked hypothermia in *Trpa1*^*+/+*^ mice, which was significantly reduced in *Trpa1*^*−/−*^ mice (n = 6). Data are mean ± sem; ^*^p < 0.05, ^**^p < 0.01, ^***^p < 0.001 compared to vehicle and ^†^p < 0.05, ^††^p < 0.01, ^†††^p < 0.001 compared to *Trpa1*^*+/+*^.

We monitored the core body temperature in response to pharmacological treatments in mice fitted with Bio-thermo iDENTICHIPS. The basal body temperature was not significantly different between naïve wildtype and *Trpa1*^*−/−*^ mice (*Trpa1*^*+/+*^ 37.8 ± 0.1°C, range 37.3–38.3; *Trpa1*^*−/−*^ 37.7 ± 0.1 °C, range 37.5–38.2, p > 0.05, n = 12 groups of 6 for each genotype). Subcutaneous (s.c.) injections of APAP evoked a time- and dose-dependent hypothermia in C57BL/6J mice (Fig. 1A). This hypothermic response reached a mean amplitude of −4.6  ±  0.4 °C 60 min after administration of APAP (300 mg/kg, s.c., n = 14 independent experiments, each with n ≥ 6). Importantly, when we compared the hypothermic activity of APAP in *Trpa1*^*+/+*^ and *Trpa1*^*−/−*^ mice, we found that APAP had no effect on body temperature in *Trpa1*^*−/−*^ mice (Fig. 1B). The antinociceptive activity of APAP is mediated by activation of TRPA1 channels expressed in the central terminals of sensory neurons^3^. However, intrathecal injection of 100 μg APAP, a dose that elicit a marked analgesia^3^, did not alter body temperature (Fig. 1C). The absence of hypothermia after intrathecal injections of analgesic doses of APAP also provides evidence that hypothermia does not contribute to the measured spinal analgesic activity of APAP in rodents.

We investigated the contribution of peripheral sensory neurons to the APAP evoked hypothermia by chemically ablating the TRPV1 expressing neurons with resiniferatoxin (RTX). Subcutaneous administration of RTX (6.3 μg/kg) evoked a profound long-lasting hypothermia in both wild-type and *Trpa1*^*−/−*^ mice, with body temperature returning to normal after about 24 h (Fig. 1D). Subsequent treatment with APAP failed to evoke a hypothermic response in RTX-treated wildtype mice and vehicle- and RTX-treated *Trpa1*^*−/−*^ mice (Fig. 1E). This observation demonstrates that both nociceptive sensory neurons and TRPA1 are necessary for the hypothermic response to stimulation with APAP. Earlier investigations proposed that APAP acts by inhibiting either COX-1 or COX-2^14^,^15^. However, pretreatment with the nonselective COX inhibitor indomethacin (10 mg/kg, s.c.) did not alter the body temperature in mice and also failed to influence the hypothermia evoked by APAP, (Fig. 1F), demonstrating that APAP acts independently of COX-1 and COX-2.

Since our earlier investigations identified activation of TRPA1 by NAPQI formed from APAP as an analgesic mechanism of action^3^, we examined whether the APAP induced hypothermia could be prevented by treatment with a pharmacological inhibitor of TRPA1. Treatment with the selective TRPA1 inhibitor CHEM5861528 (40–300 mg/kg, p.o.) had no acute effect on body temperature in wild-type mice (vehicle 37.8 ± 0.1 °C, n = 18; CHEM5861528 40 mg/kg 37.8 ± 0.1 °C, n = 6; 100 mg/kg 38.0 ± 0.1 °C, n = 6; 300 mg/kg 37.7 ± 0.2 °C, n = 6; p > 0.05) but pre-treatment with CHEM5861528 produced a dose-dependent inhibition of the APAP induced hypothermia. At the highest dose tested the effects of APAP were essentially abolished (Fig. 1G).

APAP is metabolized to NAPQI and benzoquinone which are both TRPA1 agonists. We therefore examined if other TRPA1 agonists evoke hypothermia. Cinnamaldehyde (125 mg/kg) was administered intraperitoneally to *Trpa1*^*−/−*^ and wild-type mice. A large reduction in body temperature was noted in wild-type mice, and this was greatly reduced, although not abolished, in the *Trpa1*^*−/−*^ mice (Fig. 1H).

### Involvement of TRPV1

**Figure 2:**
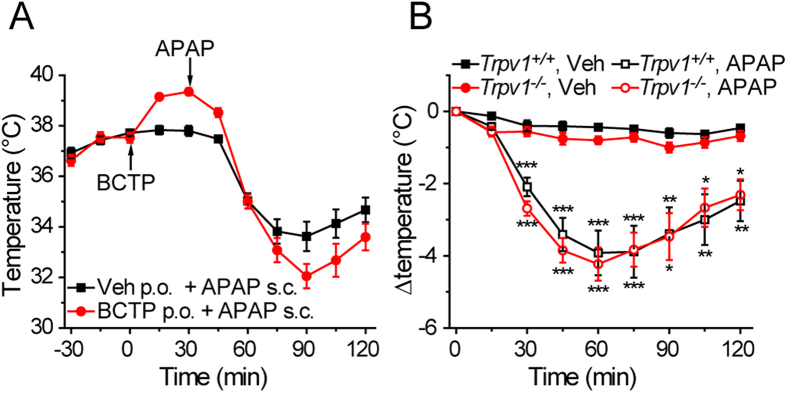
APAP induces hypothermia independently of TRPV1. (**A**) Oral administration of the TRPV1 antagonist BCTP (30 mg/kg) elicited hyperthermia in C57BL/6J mice (n = 6), but did not inhibit the hypothermia produced by a subsequent dose of APAP (300 mg/kg). (**B**) APAP (300 mg/kg) evoked identical hypothermia in *Trpv1*^*+/+*^ and *Trpv1*^*−/−*^ mice (n = 6). Data are mean ± sem; ^*^p < 0.05, ^**^p < 0.01, ^***^p < 0.001 compared to vehicle (ANOVA followed by Tukey’s HSD test).

Since TRPV1 inhibition evokes hyperthermia^16^,^17^ and TRPV1 activation elicits hypothermia, we re-evaluated the effects of APAP in wild-type mice treated with the TRPV1 antagonist BCTP^18^, and in *Trpv1*^*−/−*^ mice. The basal body temperature was not different in *Trpv1*^*+/+*^ *and Trpv1*^*−/−*^ mice (Trpv1^+/+^ 37.8 ± 0.1 °C; Trpv1^*−/−*^ 37.8 ± 0.1 °C, p > 0.2, n = 11 groups of 6 for each genotype). BCTP (30 mg/kg p.o.) evoked a small degree of hyperthermia in *Trpv1*^*+/+*^ mice but did not inhibit the hypothermia evoked by subsequent administration of APAP (Fig. 2A). In fact the reduction in body temperature was larger in mice treated with the TRPV1 antagonist, since APAP reduced the body temperature to the same level in mice treated with BCTP or vehicle. Similarly APAP exerted a robust hypothermic effect in *Trpv1*^*−/−*^ mice that was at least as large as that observed in wild-type littermates (Fig. 2B). These observations confirm the results of Ayoub *et al.*^7^, which demonstrated that TRPV1 is not involved in APAP evoked hypothermia.

### Effects on yeast-induced fever

**Figure 3:**
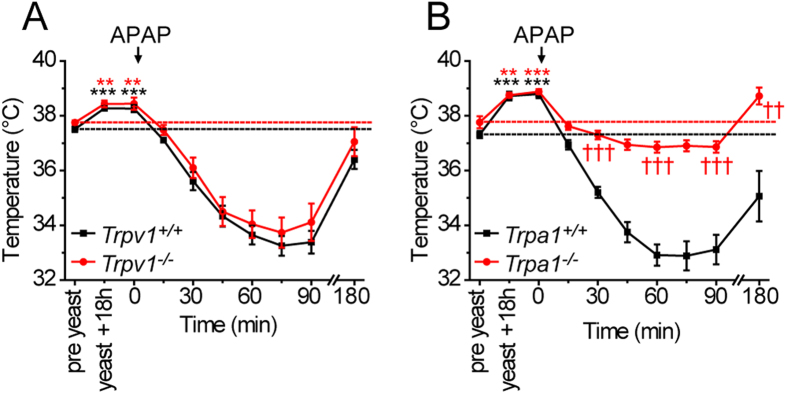
Antipyretic and hypothermic activity of APAP in yeast induced hyperthermia. Administration of yeast (2 g/kg) produced hyperthermia in wildtype, *Trpv1*^*−/−*^ and *Trpa1*^*−/−*^ mice. (**A**) APAP (300 mg/kg) produced hypothermia in both hyperthermic *Trpv1*^*+/+*^ and *Trpv1*^*−/−*^ mice (n = 8). (**B**) APAP produced hypothermia in *Trpa1*^*+/+*^ mice, but acted as an antipyretic, restoring normal, pre-yeast injection, temperature in *Trpa1*^*−/−*^ mice. Dashed lines indicate pre-yeast body temperature. Data are mean ± sem; ^*^p < 0.05, ^**^p < 0.01, ^***^p < 0.001 compared to pre-yeast injection and ^††^p < 0.01, ^†††^p < 0.001 compared to *Trpa1*^*+/+*^ (ANOVA followed by Tukey’s HSD test). For clarity, some symbols indicating statistical significance have been omitted from (**B**).

We next investigated the importance of TRPA1 or TRPV1 for the antipyretic activity of APAP. Pyrexia was induced by subcutaneous administration of yeast (2 g/kg in saline). Such a treatment resulted in an increase in body temperature of approximately 1 °C in wild-type mice and in mice lacking functional TRPV1 or TRPA1 (Fig. 3). This differs from the findings of Iida, *et al.*^19^ who found a reduced hyperthermia in *Trpv1*^*−/−*^ mice after intraperitoneal injection of lipopolysaccharide. Subsequent administration of APAP reduced body temperature and produced a robust hypothermia in both wildtype and *Trpv1*^*−/−*^ mice (Fig. 3A). In contrast, while the strong hypothermic effect of APAP was absent in *Trpa1*^*−/−*^ mice, the anti-pyretic effect of APAP was still evident, demonstrating that APAP produces antipyretic and hypothermic effects through distinct and independent mechanisms (Fig. 3B).

**Figure 4:**
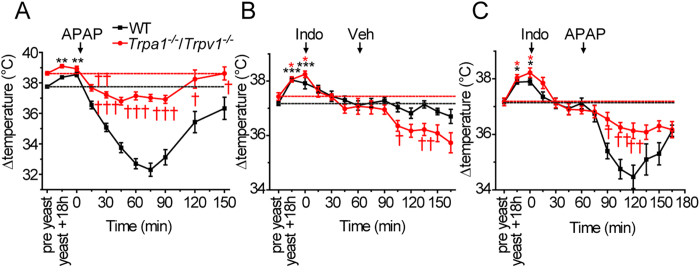
Effect of APAP and indomethacin in hyperthermic *Trpa1*^*−/−*^*/Trpv1*^*−/−*^ mice. (**A**–**C**) Administration of yeast (2 g/kg) produced hyperthermia in wildtype and *Trpa1*^*−/−*^/*Trpv1*^*−/−*^ mice. (**A**) APAP (300 mg/kg) evoked hypothermia in wildtype mice, but only restored normal body temperature in *Trpa1*^*−/−*^/*Trpv1*^*−/−*^ mice (n = 6). (**B**) The antipyretic indomethacin (30 mg/kg) restored pre-yeast injection temperature in wildtype and *Trpa1*^*−/−*^/*Trpv1*^*−/−*^ mice (n = 6). In *Trpa1*^*−/−*^/*Trpv1*^*−/−*^ mice, this was later followed by a slow reduction in body temperature. (**C**) Adminstration of APAP (300 mg/kg) 1h after indomethacin (30 mg/kg) rapidly elicited hypothermia in wildtype, but not *Trpa1*^*−/−*^/*Trpv1*^*−/−*^ mice (n = 6). The slow reduction in body temperature in *Trpa1*^*−/−*^/*Trpv1*^*−/−*^ mice was not different from that seen in (**B**) in animals receiving vehicle instead of APAP. Data are mean ± sem; *p < 0.05, **p < 0.01, ***p < 0.001 compared to pre-yeast injection and ^†^p < 0.05, ^††^p < 0.01, ^†††^p < 0.001 compared to *Trpa1+/+* (ANOVA followed by Tukey’s HSD test).

As it is possible that the anti-pyretic effect of APAP observed in *Trpa1*^*−/−*^ mice was due to an action on TRPV1^4^,^5^,^20^, we also examined the effects of APAP in yeast treated mice lacking both TRPV1 and TRPA1 (Fig. 4A). The basal body temperature of *Trpa1*^*−/−*^/*Trpv1*^*−/−*^ mice was slightly, but not significantly, higher than in matched wildtype control groups (*Trpa1*^*+/+*^/*Trpv1*^*+/+*^ 37.5 ± 0.2, range 36.9–37.9; *Trpa1*^*−/−*^/*Trpv1*^*−/−*^ 37.9 ± 0.3, range 37.1–38.6, p = 0.19, n = 7 groups of 6 mice of each genotype). Yeast administration evoked a hyperthermic response in double knockout (*Trpa1*^*−/−*^/*Trpv1*^*−/−*^) mice that was similar in magnitude to that seen in wild-type mice. Subsequent administration of APAP reduced body temperature to normal levels indicating that the anti-pyretic effects of APAP were still operational in these double knockout mice.

APAP has been proposed to exert some of its actions through inhibition of cyclo-oxygenase enzymes, despite displaying a relatively modest affinity as a COX inhibitor compared to those of NSAIDs^14^,^15^. To determine whether the antipyrexia, unlike the hypothermia, can be explained by APAP inhibition of COX, we examined the effect of APAP and indomethacin in *Trpa1*^*−/−*^/*Trpv1*^*−/−*^ mice made hyperthermic by yeast administration. Wildtype and *Trpa1*^*−/−*^/*Trpv1*^*−/−*^ mice were injected with indomethacin (10 mg/kg, s.c.) 18 h after administration of yeast and 1 hour before administration of vehicle or APAP (Fig. 4B,C). Indomethacin reduced body temperature by approximately 1 °C in both wild-type and *Trpa1*^*−/−*^/*Trpv1*^*−/−*^ mice thereby reversing the pyrexia. Subsequent administration of APAP evoked the typical hypothermia in wild-type mice but had no significant effect on body temperature in *Trpa1*^*−/−*^/*Trpv1*^*−/−*^ mice. In the double knockout mice a small further reduction in temperature was noted after APAP treatment (Fig. 4C), but a similar reduction was noted in the vehicle treated group (Fig. 4B), indicating that this small hypothermic effect is independent of APAP but may instead be caused by the continued action of indomethacin.

### Is TRPA1 responsible for the hypothermic effects of other agents?

A number of other hypothermic agents have been reported to act as TRPA1 agonists. These include some cannabinoid receptor ligands, morphine and isoflurane.

**Figure 5:**
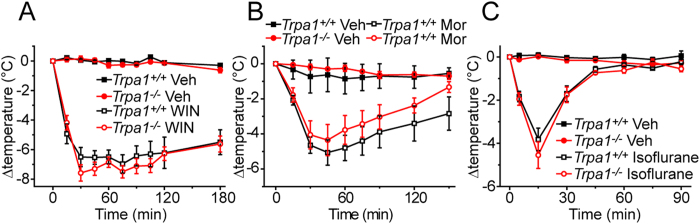
Hypothermic effects of other agents. The CB1 and CB2 receptor agonist WIN55,212-2 (**A**), the opioid receptor agonist morphine (**B**) and the general anaesthetic isoflurane (**C**) produced a marked hypothermia in *Trpa1*^*+/+*^ and *Trpa1*^*−/−*^ mice (n = 6).

A reduction in core body temperature is a well known response to cannabinoid receptor 1 (CB1) agonists^21^,^22^,^23^ and several naturally occurring and synthetic CB1 agonists have been shown to be TRPA1 agonists^3^,^24^,^25^,^26^,^27^. We therefore compared the effects of WIN55,212-2 on body temperature in *Trpa1*^*−/−*^ mice and wild-type littermates. WIN55,212-2 (3 mg/kg s.c.) evoked similar reductions (6–7 °C) in body temperature in both genotypes that lasted for at least 3 hours (Fig. 5A).

Morphine can lower body temperature in a number of species, notably at higher doses^28^ and at high concentrations acts as a TRPA1 agonist^29^. Sub-cutaneous administration of 10 mg/kg morphine lowered the body temperature of wild-type mice by 5 °C after 45–60 minutes and the amplitude and duration of the hypothermic response was not significantly different in *Trpa1*^*−/−*^ mice (Fig. 5B).

Isoflurane and other irritant general anaesthetics act as TRPA1 agonists^30^,^31^ and produce hypothermia in humans and rodents^32^,^33^,^34^,^35^. We therefore examined body temperature during isoflurane anaesthesia in wild-type and *Trpa1*^*−/−*^ mice. Isoflurane elicited a similar hypothermia of short duration in both genotypes (Fig. 5C), consistent with a TRPA1 independent mechanism.

## Discussion

The mechanisms responsible for the analgesic and antipyretic actions of APAP have been the subject of investigation for many decades. Recent studies have concluded that APAP metabolites acting on TRP channels are important for the analgesic actions of the drug. Accordingly, APAP analgesia has been ascribed to AM404 activation of TRPV1 in the central nervous system^4^,^5^,^20^ and activation of TRPA1 in the spinal cord by local formation of the electrophilic NAPQI^3^. Other studies have suggested that the cannabinoid receptor system contributes to APAP analgesia^36^,^37^. The antipyretic and hypothermic actions of APAP are not well understood. However, the findings that the hypothermic effects of APAP were retained in *Trpv1*^*−/−*^ mice as well as in mice lacking cannabinoid receptor 1 rule out a role for these pathways in APAP-evoked hypothermia^7^.

Our studies demonstrated that APAP produced a profound hypothermia in wild-type mice that was dependent on the presence of functional TRPA1 channels. APAP-evoked hypothermia was absent in *Trpa1*^*−/−*^ mice and inhibited by prior systemic treatment with a TRPA1 antagonist. The locus of hypothermic action is likely to be TRPA1 expressed in peripheral sensory neurons, since the response could be abolished by RTX mediated chemical ablation of TRPV1 containing sensory neurons. Body temperature is controlled by peripheral sensory input to the CNS as well as by central thermoregulatory mechanisms^9^,^38^,^39^. Recent studies on TRPV1 antagonists have highlighted the importance of TRPV1 expressing peripheral sensory nerves for temperature regulation. Stimulation of TRPV1 with systemic capsaicin treatment leads to hypothermia^9^,^38^, while administration of many (but not all) TRPV1 antagonists causes hyperthermia^12^,^40^,^41^,^42^. Functional ablation of these peripheral sensory neurons by prior administration of the ultrapotent TRPV1 agonist RTX results in a loss of TRPV1 mediated temperature changes^40^.

In our studies the hypothermic effects of APAP were abolished by a similar systemic treatment with RTX. However, the effect of APAP could not be ascribed to TRPV1 agonism as hypothermia was retained in *Trpv1*^*−/−*^ mice and unaffected by systemic administration of a dose of a TRPV1 antagonist that exerts a maximal analgesic effect^18^, in agreement with previous studies^7^. TRPA1 is predominantly expressed in TRPV1 containing sensory neurons^43^. The functional ablation of the TRPV1-expressing neurons by RTX will therefore have severely compromised or abolished TRPA1 responses, explaining the very marked effect of RTX treatment on APAP evoked temperature changes.

Intraperitoneal administration of the TRPA1 agonist cinnamaldehyde produced a significant hypothermia in wild-type mice that was greatly reduced, although not completely abolished, in *Trpa1*^*−/−*^ mice. This finding demonstrates that TRPA1 agonism can influence body temperature, almost certainly because TRPA1 is co-localized in TRPV1 containing thermoregulatory sensory nerves. In addition, it is consistent with the hypothesis that activation of TRPA1 by electrophilic APAP metabolites is responsible for the observed hypothermia. Electrophilic APAP metabolites are formed in the liver and liver afferent neurons are therefore prime candidates for the site of action. Importantly, we found that genetic or pharmacological blockade of TRPA1 does not produce hyperthermia in rodents, in agreement with an earlier report^44^. This observation indicates that TRPA1, unlike TRPV1, does not have a significant tonic activity in thermosensitive afferent fibres under normal conditions^12^.

Although TRPA1 is important for the hypothermic effects of APAP, our studies suggest that other mechanisms contribute to its antipyretic effects. Yeast induced hyperthermia was of a similar magnitude in wild-type, *Trpa1*^*−/−*^ and *Trpa1*^*−/−*^*/Trpv1*^*−/−*^ (double knockout) mice. The absence of APAP evoked hypothermia in both *Trpa1*^*−/−*^ and *Trpa1*^*−/−*^*/Trpv1*^*−/−*^ mice allowed the antipyretic effects to be studied in isolation. APAP reduced the elevated body temperature to normal levels in *Trpa1*^*−/−*^ and *Trpa1*^*−/−*^*/Trpv1*^*−/−*^ mice, indicating that neither TRP channel plays a role in the antipyretic actions of APAP.

We explored the possibility that the antipyretic effect of APAP was mediated by cyclo-oxygenase inhibition by examining the effect of APAP in mice pre-treated with indomethacin. In wild-type and *Trpa1*^*−/−*^*/Trpv1*^*−/−*^ mice, indomethacin reversed the pyrexia and normalized body temperature to the pre-yeast levels, indicating that these TRP channels are not involved in the antipyretic effects of cyclo-oxygenase inhibitors. Subsequent APAP administration evoked hypothermia in wild-type mice, which demonstrated that cyclo-oxygenase activity is not important for this effect of APAP. In indomethacin pre-treated *Trpa1*^*−/−*^*/Trpv1*^*−/−*^ mice, a continued small slow reduction in body temperature was noted after administration of either APAP or vehicle. The lack of any obvious APAP induced temperature change when the cyclo-oxygenase enzymes are already inhibited could be interpreted as evidence that cyclo-oxygenase inhibition underlies the antipyretic effects of APAP. However, it is possible that APAP had no effect because the pyrexia had been reversed and body temperature normalized by the indomethacin treatment.

Although the TRPA1 agonist cinnamaldehyde evoked TRPA1-dependent hypothermia, a range of other hypothermic agents that have been reported to act as TRPA1 agonists retained the ability to reduce body temperature in *Trpa1*^*−/−*^ mice. These included the general anaesthetic isoflurane, the cannabinoid receptor 1 agonist WIN55,212-2 and the opioid receptor agonist morphine. Despite the common property of TRPA1 agonism, these diverse agents must reduce body temperature primarily by other mechanisms at the doses used in our experiments.

## Conclusions

Our results demonstrate that activation of TRPA1 in sensory neurons mediates the hypothermic effect of APAP in mice. These findings support our previous identification of TRPA1 as a target for the analgesic activity of APAP^3^. Importantly, APAP does not reduce body temperature after intrathecal injections, demonstrating that hypothermia is not required for its spinal antinociceptive effect. The hypothermic effect of APAP is completely lost in *Trpa1*^*−/−*^ mice and resistant to inhibition of cyclo-oxygenase. However, it is possible that APAP exerts its antipyretic activity by cyclo-oxygenase inhibition, since APAP reversed yeast induced hyperthermia in mice lacking both TRPA1 and TRPV1. The TRPA1 mediated hypothermia may explain the improved antipyretic activity seen with combinations of APAP and the NSAID ibuprofen^2^.

## Methods

### Animals

All animal procedures were carried out in accordance with the UK Animals (Scientific Procedures) Act 1986. All experimental procedures were approved by the King’s College London Animal Care and Ethics Committee. Data shown are from female C57BL/6J mice or from male and female homozygote *Trpa1*^*−/−*^ and *Trpa1*^+/+^ littermates. C57BL/6J mice were obtained from Charles River UK. TRPA1-null mice and wild-type littermates were bred from heterozygous mice provided by Drs Kelvin Kwan and David Corey (Harvard Medical School, Boston, MA). TRPV1–null mice were bred from homozygous mice obtained from the Jackson Laboratory (Bar Harbor, ME). *Trpa1*^*−/−*^/*Trpv1*^*−/−*^ mice were generated in house by crossing the *Trpa1*^*−/−*^ and *Trpv1*^*−/−*^ mice.

### Temperature measurements

Under general anaesthesia, the lower back of the mice was shaved. The mice were then implanted subcutaneously in the shoulder region with a small identification chip incorporating a quick reading thermometer accurate to 0.1 degree °C (idENTICHIP® with Bio-Thermo™, AnimalCare Ltd. UK). The small skin incision was closed using tissue adhesive (Vetbond, 3M).

Bio-Thermo chips have many advantages over other commonly used methods for recording body temperature. Body temperatures can be measured repeatedly without disturbing the animals using a hand-held scanner. As each chip has a unique identity, they enable animals to be group housed in their home cages. It is less stressful than rectal temperature recording and is cheaper than radiotelemetry methods.

During each experiment, body temperature was recorded every 15 min for up to 45 min prior to drug administration to establish baseline temperatures and then post dose for up to 24 h.

### Drugs

Acetaminophen (APAP, 4-Acetamidophenol; Sigma-Aldrich, UK) and morphine (Sigma-Aldrich, UK) were prepared in 0.9% saline (with warming for APAP). WIN55,212-2 (Tocris, UK) was made up in 5% DMSO/5% Tween 80/0.9% saline, RTX (Sigma-Aldrich, UK) in 10% ethanol/10% Tween 80/saline, indomethacin (Sigma-Aldrich, UK) in 5% sodium hydrogen carbonate (pH to 7.4), cinnamaldehyde (Fluka) in 0.5% Tween 80/saline and brewer’s yeast; (Acros Organics) in 0.9% saline. BCTP (7-tert-butyl-6-(4-chloro-phenyl)-2-thioxo-2,3-dihydro-1H-pyrido[2,3-d]pyrimidin-4-one) was suspended in 0.5% methyl cellulose. CHEM5861528 (Chembridge Corporation, USA), capsaicin (Sigma-Aldrich, UK) was administered in 10% DMSO/saline. Mice were exposed to isoflurane (Abbott Industries Ltd. UK) at 2.5% in 95%O_2_/5% CO_2_ for 5 minutes.

### Data analysis

All data are presented as the mean ± sem. Plots show the change in body temperature from baseline readings or the raw recorded temperature. Statistical analysis was carried out on raw data using repeated measures ANOVA followed by post-hoc analysis using Tukey’s HSD test (p < 0.05 was set as the level of statistical significance).

## Additional Information

**How to cite this article**: Gentry, C. *et al.* TRPA1 mediates the hypothermic action of acetaminophen. *Sci. Rep.* **5**, 12771; doi: 10.1038/srep12771 (2015).

## References

[b1] Derry C. J., Derry S. & Moore R. A. Single dose oral ibuprofen plus paracetamol (acetaminophen) for acute postoperative pain. The Cochrane database of systematic reviews 6, CD010210, 10.1002/14651858.CD010210.pub2 (2013). 23794268 PMC6485825

[b2] Wong T. *et al.* Combined and alternating paracetamol and ibuprofen therapy for febrile children. The Cochrane database of systematic reviews 10, CD009572, 10.1002/14651858.CD009572.pub2 (2013). 24174375 PMC6532735

[b3] Andersson D. A. *et al.* TRPA1 mediates spinal antinociception induced by acetaminophen and the cannabinoid Delta(9)-tetrahydrocannabiorcol. Nat Commun 2, 551, 10.1038/ncomms1559 (2011). 22109525

[b4] Hogestatt E. D. *et al.* Conversion of acetaminophen to the bioactive N-acylphenolamine AM404 via fatty acid amide hydrolase-dependent arachidonic acid conjugation in the nervous system. J Biol Chem 280, 31405–31412 (2005). 15987694 10.1074/jbc.M501489200

[b5] Mallet C. *et al.* TRPV1 in brain is involved in acetaminophen-induced antinociception. PLoS One 5, 10.1371/journal.pone.0012748 (2010). PMC2941447 20862299

[b6] Li S. *et al.* Acetaminophen: antipyretic or hypothermic in mice? In either case, PGHS-1b (COX-3) is irrelevant. Prostaglandins & other lipid mediators 85, 89–99, 10.1016/j.prostaglandins.2007.10.007 (2008). 18083054 PMC2329595

[b7] Ayoub S. S. *et al.* Paracetamol-induced hypothermia is independent of cannabinoids and transient receptor potential vanilloid-1 and is not mediated by AM404. Drug metabolism and disposition: the biological fate of chemicals 39, 1689–1695, 10.1124/dmd.111.038638 (2011). 21628499

[b8] den Hertog H. M. *et al.* The Paracetamol (Acetaminophen) In Stroke (PAIS) trial: a multicentre, randomised, placebo-controlled, phase III trial. Lancet Neurol 8, 434–440, 10.1016/S1474-4422(09)70051-1 (2009). 19297248

[b9] Jancso-Gabor A., Szolcsanyi J. & Jancso N. Irreversible impairment of thermoregulation induced by capsaicin and similar pungent substances in rats and guinea-pigs. The Journal of physiology 206, 495–507 (1970). 5498502 10.1113/jphysiol.1970.sp009027 PMC1348662

[b10] Miller M. S., Brendel K., Buck S. H. & Burks T. F. Dihydrocapsaicin-induced hypothermia and substance P depletion. Eur J Pharmacol 83, 289–292 (1982). 6184240 10.1016/0014-2999(82)90263-1

[b11] Woods A. J., Stock M. J., Gupta A. N., Wong T. T. & Andrews P. L. Thermoregulatory effects of resiniferatoxin in the rat. Eur J Pharmacol 264, 125–133 (1994). 7851474 10.1016/0014-2999(94)00445-5

[b12] Gavva N. R. *et al.* The vanilloid receptor TRPV1 is tonically activated *in vivo* and involved in body temperature regulation. J Neurosci 27, 3366–3374 (2007). 17392452 10.1523/JNEUROSCI.4833-06.2007 PMC6672109

[b13] Chen J. *et al.* Selective blockade of TRPA1 channel attenuates pathological pain without altering noxious cold sensation or body temperature regulation. Pain 152, 1165–1172, 10.1016/j.pain.2011.01.049 (2011). 21402443

[b14] Mitchell J. A., Akarasereenont P., Thiemermann C., Flower R. J. & Vane J. R. Selectivity of nonsteroidal antiinflammatory drugs as inhibitors of constitutive and inducible cyclooxygenase. Proc Natl Acad Sci USA 90, 11693–11697 (1993). 8265610 10.1073/pnas.90.24.11693 PMC48050

[b15] Flower R. J. & Vane J. R. Inhibition of prostaglandin synthetase in brain explains the anti-pyretic activity of paracetamol (4-acetamidophenol). Nature 240, 410–411 (1972). 4564318 10.1038/240410a0

[b16] Gavva N. R. *et al.* Repeated administration of vanilloid receptor TRPV1 antagonists attenuates hyperthermia elicited by TRPV1 blockade. J Pharmacol Exp Ther 323, 128–137 (2007). 17652633 10.1124/jpet.107.125674

[b17] Gavva N. R. *et al.* Pharmacological blockade of the vanilloid receptor TRPV1 elicits marked hyperthermia in humans. Pain 136, 202–210, 10.1016/j.pain.2008.01.024 (2008). 18337008

[b18] Nash M. S. *et al.* 7-tert-Butyl-6-(4-chloro-phenyl)-2-thioxo-2,3-dihydro-1H-pyrido[2,3-d]pyrimidin-4 -one, a classic polymodal inhibitor of transient receptor potential vanilloid type 1 with a reduced liability for hyperthermia, is analgesic and ameliorates visceral hypersensitivity. J Pharmacol Exp Ther 342, 389–398, 10.1124/jpet.112.191932 (2012). 22566669

[b19] Iida T., Shimizu I., Nealen M. L., Campbell A. & Caterina M. Attenuated fever response in mice lacking TRPV1. Neurosci Lett 378, 28–33, 10.1016/j.neulet.2004.12.007 (2005). 15763167

[b20] Barriere D. A. *et al.* Fatty acid amide hydrolase-dependent generation of antinociceptive drug metabolites acting on TRPV1 in the brain. PLoS One 8, e70690, 10.1371/journal.pone.0070690 (2013). 23940628 PMC3734263

[b21] Compton D. R., Gold L. H., Ward S. J., Balster R. L. & Martin B. R. Aminoalkylindole analogs: cannabimimetic activity of a class of compounds structurally distinct from delta 9-tetrahydrocannabinol. J Pharmacol Exp Ther 263, 1118–1126 (1992). 1335057

[b22] Compton D. R. *et al.* Cannabinoid structure-activity relationships: correlation of receptor binding and *in vivo* activities. J Pharmacol Exp Ther 265, 218–226 (1993). 8474008

[b23] Fox A. *et al.* The role of central and peripheral Cannabinoid1 receptors in the antihyperalgesic activity of cannabinoids in a model of neuropathic pain. Pain 92, 91–100 (2001). 11323130 10.1016/s0304-3959(00)00474-7

[b24] Akopian A. N., Ruparel N. B., Patwardhan A. & Hargreaves K. M. Cannabinoids desensitize capsaicin and mustard oil responses in sensory neurons via TRPA1 activation. J Neurosci 28, 1064–1075, 10.1523/JNEUROSCI.1565-06.2008 (2008). 18234885 PMC6671418

[b25] Jordt S. E. *et al.* Mustard oils and cannabinoids excite sensory nerve fibres through the TRP channel ANKTM1. Nature 427, 260–265 (2004). 14712238 10.1038/nature02282

[b26] De Petrocellis L. *et al.* Plant-derived cannabinoids modulate the activity of transient receptor potential channels of ankyrin type-1 and melastatin type-8. J Pharmacol Exp Ther 325, 1007–1015, 10.1124/jpet.107.134809 (2008). 18354058

[b27] Zygmunt P. M., Andersson D. A. & Hogestatt E. D. Delta 9-tetrahydrocannabinol and cannabinol activate capsaicin-sensitive sensory nerves via a CB1 and CB2 cannabinoid receptor-independent mechanism. J Neurosci 22, 4720–4727 (2002). 12040079 10.1523/JNEUROSCI.22-11-04720.2002 PMC6758782

[b28] Adler M. W., Geller E. B., Rosow C. E. & Cochin J. The opioid system and temperature regulation. Annual review of pharmacology and toxicology 28, 429–449, 10.1146/annurev.pa.28.040188.002241 (1988). 2837979

[b29] Forster A. B., Reeh P. W., Messlinger K. & Fischer M. J. High concentrations of morphine sensitize and activate mouse dorsal root ganglia via TRPV1 and TRPA1 receptors. Mol Pain 5, 17, 10.1186/1744-8069-5-17 (2009). 19371406 PMC2672059

[b30] Eilers H. *et al.* Pungent general anesthetics activate transient receptor potential-A1 to produce hyperalgesia and neurogenic bronchoconstriction. Anesthesiology 112, 1452–1463, 10.1097/ALN.0b013e3181d94e00 (2010). 20463581

[b31] Matta J. A. *et al.* General anesthetics activate a nociceptive ion channel to enhance pain and inflammation. Proc Natl Acad Sci USA 105, 8784–8789, 10.1073/pnas.0711038105 (2008). 18574153 PMC2438393

[b32] Ramachandra V., Moore C., Kaur N. & Carli F. Effect of halothane, enflurane and isoflurane on body temperature during and after surgery. Br J Anaesth 62, 409–414 (1989). 2706176 10.1093/bja/62.4.409

[b33] Smith D., Wood M., Pearson J., Mehta R. L. & Carli F. Effects of enflurane and isoflurane in air-oxygen on changes in thermal balance during and after surgery. Br J Anaesth 65, 754–759 (1990). 2265044 10.1093/bja/65.6.754

[b34] Kushikata T. *et al.* Isoflurane increases norepinephrine release in the rat preoptic area and the posterior hypothalamus *in vivo* and *in vitro*: Relevance to thermoregulation during anesthesia. Neuroscience 131, 79–86, 10.1016/j.neuroscience.2004.11.007 (2005). 15680693

[b35] Zeller A., Arras M., Jurd R. & Rudolph U. Mapping the contribution of beta3-containing GABAA receptors to volatile and intravenous general anesthetic actions. BMC Pharmacol 7, 2, 10.1186/1471-2210-7-2 (2007). 17319964 PMC1810244

[b36] Mallet C. *et al.* Endocannabinoid and serotonergic systems are needed for acetaminophen-induced analgesia. Pain 139, 190–200, 10.1016/j.pain.2008.03.030 (2008). 18485596

[b37] Dalmann R., Daulhac L., Antri M., Eschalier A. & Mallet C. Supra-spinal FAAH is required for the analgesic action of paracetamol in an inflammatory context. Neuropharmacology 91, 63–70, 10.1016/j.neuropharm.2014.11.006 (2015). 25448494

[b38] Jancso-Gabor A., Szolcsanyi J. & Jancso N. Stimulation and desensitization of the hypothalamic heat-sensitive structures by capsaicin in rats. The Journal of physiology 208, 449–459 (1970). 5500735 10.1113/jphysiol.1970.sp009130 PMC1348759

[b39] Romanovsky A. A. *et al.* The transient receptor potential vanilloid-1 channel in thermoregulation: a thermosensor it is not. Pharmacological reviews 61, 228–261, 10.1124/pr.109.001263 (2009). 19749171 PMC2763780

[b40] Steiner A. A. *et al.* Nonthermal activation of transient receptor potential vanilloid-1 channels in abdominal viscera tonically inhibits autonomic cold-defense effectors. J Neurosci 27, 7459–7468 (2007). 17626206 10.1523/JNEUROSCI.1483-07.2007 PMC6672610

[b41] Garami A. *et al.* Contributions of different modes of TRPV1 activation to TRPV1 antagonist-induced hyperthermia. J Neurosci 30, 1435–1440, 10.1523/JNEUROSCI.5150-09.2010 (2010). 20107070 PMC2824913

[b42] Gomtsyan A. *et al.* TRPV1 ligands with hyperthermic, hypothermic and no temperature effects in rats. Temperature **In press**, 10.1080/23328940.2015.1046013 (2015). PMC4843892 27227030

[b43] Story G. M. *et al.*. ANKTM1, a TRP-like channel expressed in nociceptive neurons, is activated by cold temperatures. Cell 112, 819–829 (2003). 12654248 10.1016/s0092-8674(03)00158-2

[b44] de Oliveira C. *et al.* Transient receptor potential channel ankyrin-1 is not a cold sensor for autonomic thermoregulation in rodents. J Neurosci 34, 4445–4452, 10.1523/JNEUROSCI.5387-13.2014 (2014). 24671991 PMC3965775

